# A Point-of-Care Device for Fully Automated, Fast and Sensitive Protein Quantification via qPCR

**DOI:** 10.3390/bios12070537

**Published:** 2022-07-19

**Authors:** Francesca Romana Cavallo, Khalid Baig Mirza, Sara de Mateo, Luca Miglietta, Jesus Rodriguez-Manzano, Konstantin Nikolic, Christofer Toumazou

**Affiliations:** 1 Centre for Bio-Inspired Technology, Department of Electrical and Electronic Engineering, Imperial College London, London SW7 2AZ, UK; francesca.cavallo14@imperial.ac.uk (F.R.C.); s.de-mateo-lopez@imperial.ac.uk (S.d.M.); l.miglietta@imperial.ac.uk (L.M.); konstantin.nikolic@uwl.ac.uk (K.N.); c.toumazou@imperial.ac.uk (C.T.); 2 Department of Biotechnology and Medical Engineering, National Institute of Technology, Rourkela 769008, India; baigm@nitrkl.ac.in; 3 Department of Infectious Disease, Imperial College London, London SW7 2AZ, UK; 4 School of Computing and Engineering, University of West London, London W5 5RF, UK

**Keywords:** point-of-care, noise minimisation, qPCR, algorithm, diagnostics, protein quantification, DNA aptamers

## Abstract

This paper presents a fully automated point-of-care device for protein quantification using short-DNA aptamers, where no manual sample preparation is needed. The device is based on our novel aptamer-based methodology combined with real-time polymerase chain reaction (qPCR), which we employ for very sensitive protein quantification. DNA amplification through qPCR, sensing and real-time data processing are seamlessly integrated into a point-of-care device equipped with a disposable cartridge for automated sample preparation. The system’s modular nature allows for easy assembly, adjustment and expansion towards a variety of biomarkers for applications in disease diagnostics and personalised medicine. Alongside the device description, we also present a new algorithm, which we named PeakFluo, to perform automated and real-time quantification of proteins. PeakFluo achieves better linearity than proprietary software from a commercially available qPCR machine, and it allows for early detection of the amplification signal. Additionally, we propose an alternative way to use the proposed device beyond the quantitative reading, which can provide clinically relevant advice. We demonstrate how a convolutional neural network algorithm trained on qPCR images can classify samples into high/low concentration classes. This method can help classify obese patients from their leptin values to optimise weight loss therapies in clinical settings.

## 1. Introduction

Point-of-care (POC) platforms facilitate simple detection or quantification of biomarkers without the need for specialised laboratory facilities, hence enabling easy detection, fast diagnosis and treatment [1], thus leading to better clinical outcomes for the patient. However, conventional POC devices for protein detection are based on the enzyme-linked immunosorbent assay (ELISA), which requires expensive equipment for sensitive quantitative detection. Immunoassays that are cheaper and simpler are routinely used in POC tests (common examples are the home pregnancy test and tests for hepatitis [2] or HIV detection [3]) but they cannot reach the sensitivity levels needed for the quantification of proteins in saliva, which can reach the pg/mL [4]. Aptamers, short single-strand sequences of DNA/RNA, represent a viable alternative to antibodies, as they offer extended shelf life and simple workflow [5]. For these reasons, they are especially suited for POC devices. Additionally, DNA aptamers can be quantified through real-time polymerase chain reaction (qPCR) [6], a robust and sensitive DNA quantification method with limits of detection of a few copies.

Although qPCR is mainly conducted in laboratory settings, portable, compact and cheap qPCR machines have been developed [7,8,9,10], but lack the automated sample preparation. On the other hand, Toumazou et al. [11] implemented a device for automated sample preparation with a qPCR machine that does not require laboratory facilities and trained personnel. The device was originally designed for DNA genotyping and was later adapted for the diagnosis of SARS-CoV-2 [12]. This work proposes the adaptation of this device to detect proteins in human samples through PCR amplification of target-specific aptamers. Additionally, what renders our work novel is that while several aptasensors based on qPCR have been implemented [13,14,15,16], a fully automated POC platform based on aptamers and qPCR amplification has never been developed.

In this paper, we expand on the work presented in [17] around a point-of-care device for sensitive protein detection, but compared to our previous works, here we describe the integration of our novel aptasensor (presented in [18]) with a fully automated POC device. At the basis of the POC device, there is the amplification of the chemical signal through qPCR, which allows accurate detection of minimal protein concentrations. DNA amplification through qPCR, sensing and real-time data processing are seamlessly integrated into a point-of-care device equipped with a disposable cartridge for automated sample preparation. The system’s modular nature allows for easy assembly, adjustment and expansion towards a variety of biomarkers for applications in disease diagnostics and personalised medicine. The device completely automates the workflow from sample-to-result, including fully integrated sample preparation and target identification without human intervention. Additionally, we have developed a novel custom algorithm for the real-time processing of qPCR data from aptamer-based assays. The algorithm performs automated and real-time quantification of proteins, while also enabling early signal detection.

The device has been developed to quantify the gut hormone leptin, an essential protein for the regulation of energy balance, with links to metabolic conditions such as obesity and type 2 diabetes [19]. Leptin is used in therapies for leptin-deficient patients and is a possible biomarker for obesity management therapies, since leptin resistance is related to obesity and leptin levels vary with dieting regimens [20]. In addition, leptin is a potential biomarker of Crohn’s disease, ulcerative colitis [21] and ischemic heart disease in type 2 diabetes [22]. As an example of a clinical application that would benefit from using our POC device, we have trained and validated a convolutional neural network (CNN) to classify leptin concentrations based on a specified threshold. Leptin concentrations are associated with obesity types, either characterised by leptin resistance (high leptin levels) or by leptin deficiency (low leptin levels) [23] and thus, it is crucial to know the underlying cause of the condition to decide on the most appropriate course of action; therefore, classifying the type of obesity based on circulating leptin levels could provide support to decide on the best therapy, as well as providing insights into its efficacy.

Although we use the quantification of leptin to validate this platform, the present POC device can be easily adapted to quantify any target protein; thus, this technique can be used for sensitive and precise POC antigen or antibody testing for various conditions, provided the target-specific aptamer is available and suitable adaptor and primer sequences are designed.

## 2. Background

### 2.1. Aptamer-Based POC Devices for Protein Quantification

Aptamers are in many ways more suited to POC platforms than antibodies, as their affinity and sensitivity can be extremely high, and they are more stable and robust to ambient conditions than antibodies. Since aptamers can be labelled with a fluorophore, they have been mainly used as a direct replacement of antibodies in ELISAs and biosensors [24]. Aptamer-based colourimetric and rapid lateral flow assays are routinely used in low-resource settings for food safety [25], drug abuse testing, pregnancy tests and cancer biomarkers [26]; however, they provide qualitative monitoring and are not suitable for highly sensitive applications since they rely on visual quantification.

Although aptamers have been used as direct substitutes for antibodies, aptamers allow great flexibility to develop POC platforms. Unlike antibodies, aptamers offer homogeneous assays that avoid immobilisation and washing steps [27], thus enabling quick and simple testing platforms. Another advantage of DNA/RNA aptamers is the possibility to use qPCR as the quantification method, which significantly improves sensitivity and resolution. For example, Guo et al. [13] developed an ultrasensitive assay based on aptamers and qPCR amplification for the food toxin aflatoxin B1 that achieved extremely high sensitivity (25 fg/mL). Aptasensors with immobilised aptamers and qPCR have also been developed for ochratoxin A [14] (another food contaminant); aptasensors based on rolling cycle amplification were implemented for the food contaminant Vibrio parahaemolyticus [15] and for adenosine triphosphate [16]; yet, no qPCR-based aptasensor has been designed as a fully automated POC platform before.

### 2.2. Techniques for the Analysis of qPCR Data

**Figure 1:**
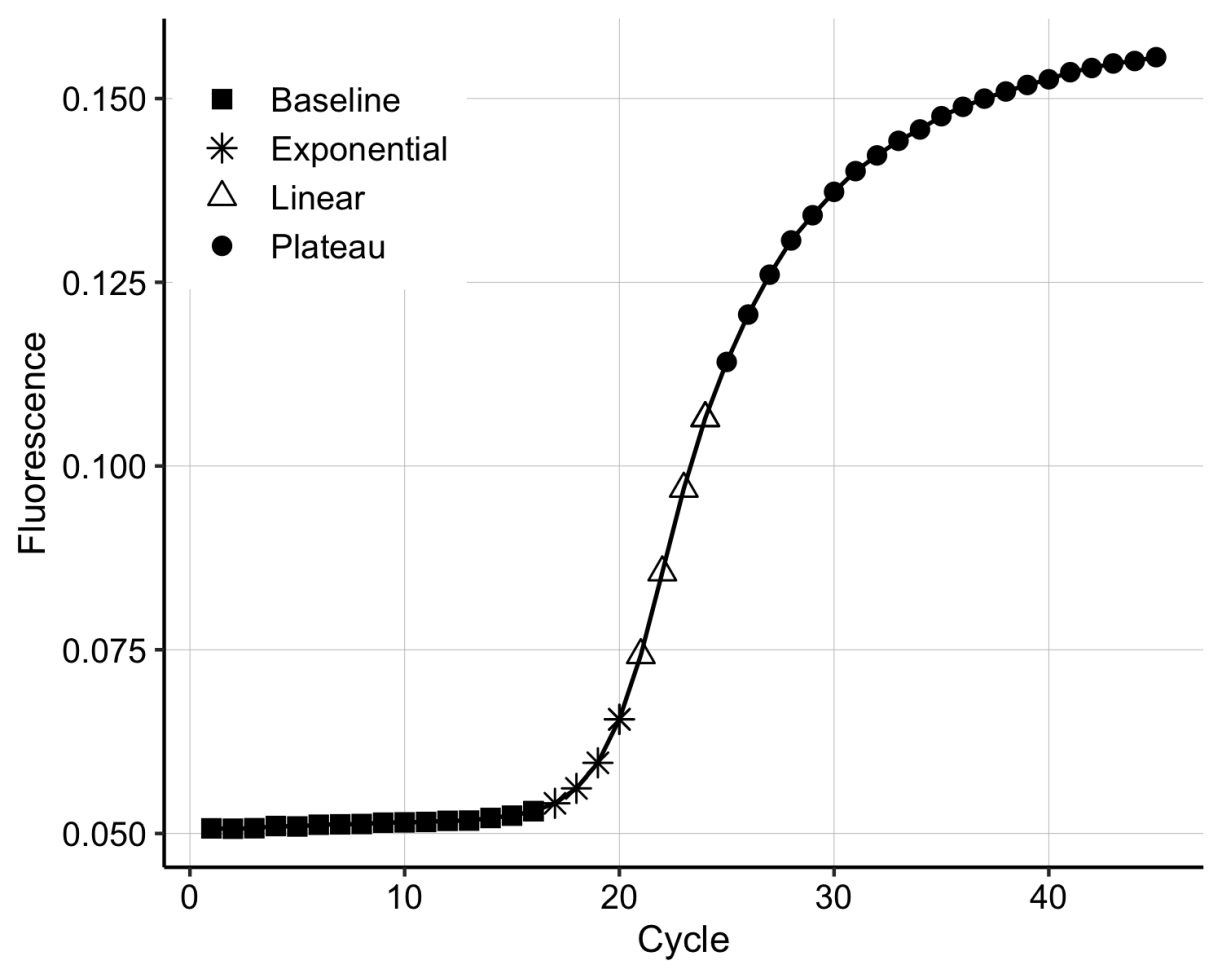
The four phases of the real-time polymerase chain reaction (qPCR) amplification curve.

Polymerase chain reaction is based on the DNA replication process that naturally occurs during cell division. qPCR has four distinct phases (Figure 1); (1) during the background phase, the target DNA is too low in concentration, and the background noise covers the signal; (2) once the target DNA becomes detectable, the output signal enters the exponential phase, in which the DNA is doubled at every cycle; (3) in the exponential region, the curve is described by the equation $y_{n}=y_{0}{\left(1+E\right)}^{n}$, where *n* is the cycle number, $y_{n}$ is the number of DNA copies at cycle *n*, $y_{0}$ is the initial amount of DNA and *E* is the reaction efficiency [28]; (4) the linear phase appears due to inefficiencies in the amplification process, such as suboptimal melting temperatures for the primers and non-specific amplification. Finally, the process enters a plateau due to the depletion of qPCR resources, such as polymerase and primers.

The $C_{t}$ or threshold cycle value is the cycle number at which the fluorescence generated within a reaction crosses the fluorescence threshold, a fluorescent signal significantly above the background fluorescence. This value is inversely proportional to the DNA concentration. The $C_{t}$ values of samples of known concentrations generate a standard curve, which is used to quantify the concentration of the targeted substance; therefore, the precision of the $C_{t}$ values is crucial.

There are numerous methods to obtain the $C_{t}$ value. One method is to set the $C_{t}$ as the cycle at which the fluorescence signal intersects a preset threshold; however, this approach is sensitive to scale and therefore, requires scaling of the data, which is usually implemented by fitting a logistic function [29]. Other methods look at the 1st or 2nd derivatives of the fluorescent curve, and the cycle where they reach the maximum is set to be the threshold $C_{t}$. More complicated algorithms also exist and they can achieve more accurate $C_{t}$ estimates [30,31], but they are unsuitable to compute on a simple POC device without an FPGA or sophisticated microcontroller, which would increase the complexity of the device.

The $C_{t}$ values can also be derived in real-time during the qPCR amplification by estimating a model at each cycle, until a threshold is reached, after which the qPCR is interrupted. Han et al. [32] have demonstrated that this method can achieve high accuracy and significantly reduce the number of cycles needed for quantification; however, due to the requirement to estimate a new model at every cycle, this method is also computationally expensive and therefore not suited to a simple and inexpensive POC device.

Alternatives to the $C_{t}$ method exist for the absolute quantification of DNA. The $C_{y0}$ approach [31], fits a sigmoid to the amplification curve and takes $C_{y0}$ as the intersection between the abscissa axis and the tangent of the inflection point. Another method fits the sigmoid up to a “cutoff cycle” and takes $F_{0}$ as the fluorescence at cycle 0 [33]. Finally, Moniri et al. [34] combined the aforementioned methods to generate a multidimensional standard curve that optimizes qPCR performance by employing multiple features and automatically detecting outliers.

## 3. Material and Methods

**Figure 2:**
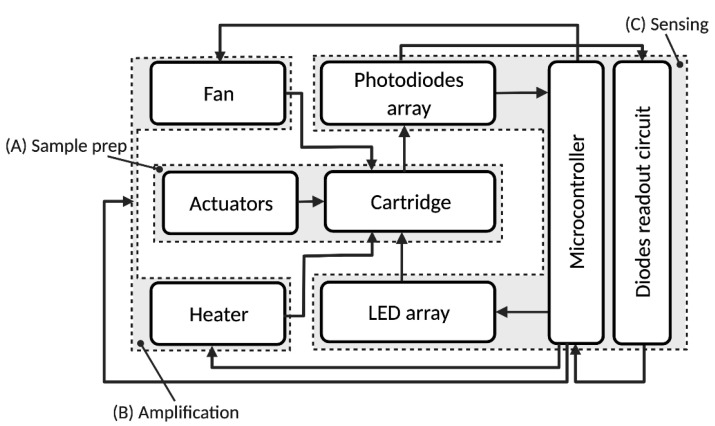
Block diagram of the protein quantification system. The different functional blocks are: (A) sample preparation; (B) thermocycler/DNA amplification; (C) sensing/data processing. Reprinted/adapted with permission from Ref. [17]. 2021, IEEE.

**Figure 3:**
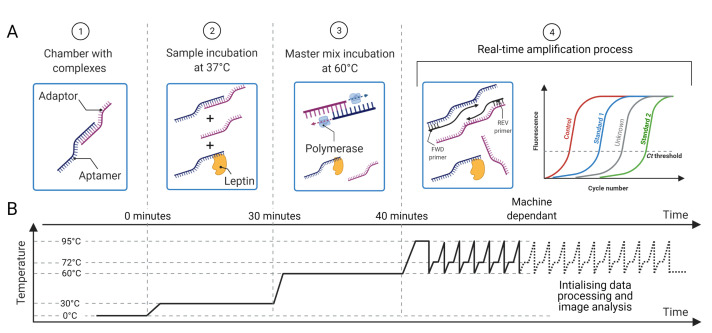
(**A**) Proposed methodology for protein sensing: (1) the chambers are preloaded with aptamer–adaptor complexes; (2) the sample containing the target protein (leptin) is loaded and the protein displaces the complexes proportionally to its concentration; (3) the master mix with the enzyme for complex elongation and qPCR is added; (4) the primers are loaded and the qPCR that amplifies the leftover elongated complexes starts. (**B**) Temperature profile for sample preparation and qPCR, which is preset in the machine. The time taken to quantify the sample is machine dependant as it depends on the amount of aptamer–adaptor complexes formed and retained during the sample preparation steps 1–4. Reprinted/adapted with permission from Ref. [17]. 2021, IEEE.

As shown in Figure 2, the system is composed three main modules: a sample preparation module (A), a thermocycler/DNA amplification module (B) for qPCR and a sensing and data processing module (C). The system automates the aptamer-based quantification method depicted in Figure 3 and in our previous publication [18], which is briefly described in the next section.

### 3.1. Sequences and Primer Design

The sequences used in the experiments are presented in Table 1. The leptin aptamer is from Ashley and Li [35] and the adaptor is designed to have 100 bp from the Green Fluorescent Protein (GFP) gene with an extra 26 bp for aptamer binding. The primers were designed using GENEious Prime ( https://www.geneious.com, accessed on 19 July 2022) and the $T_{m}$ of the amplification product was determined by the Melting Curve Predictions Software (uMELT) package (https://www.dna-utah.org/umelt/quartz/um.php, accessed on 19 July 2022). Primer specificity was assessed by melting curve analysis after RT-PCR, which consisted of 1 cycle at 95 ${}^{\circ }$C for 1 min, 40 ${}^{\circ }$C for 2 min followed by a continuous increase in temperature to 90 ${}^{\circ }$C at the rate of 0.5 ${}^{\circ }$C per second.

### 3.2. Aptamer-Adaptor Complex Methodology

The aptamer-based method for ultrasensitive protein quantification is described in detail in [18] and briefly presented here. The aptamer and the adaptor are single-stranded DNA, each one being complementary to the other so that they are loosely bound by complementary base pair bonding to form a complex. To form such complexes, adaptors and aptamers are incubated for 15 min at 37 ${}^{\circ }$C. Then, the leptin sample is added to the complexes, which are displaced by the leptin molecules breaking the aptamer–adaptor bond and hybridising to the aptamers; therefore, the aptamer selectively disassociates from the nucleic acid to selectively bind to leptin in the sample. The solution is then incubated with the enzyme polymerase for 10 min at 60 ${}^{\circ }$C, which elongates the leftover complexes, thus turning them into double-stranded DNA ready for qPCR amplification. A graphical description of the protocol is presented in Figure 3.

### 3.3. Cartridge

**Figure 4:**
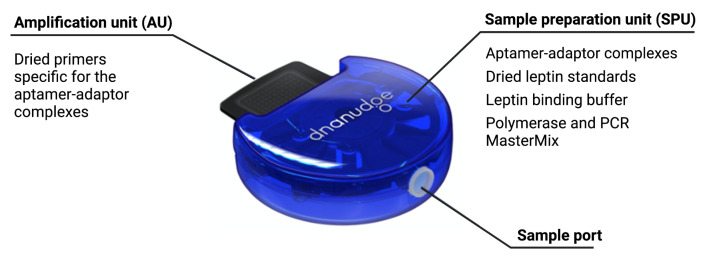
Cartridge, which consists of two main units: the sample preparation unit (SPU) and amplification unit (AU). Reprinted/adapted with permission from Ref. [12]. 2020, the Authors.

**Figure 5:**
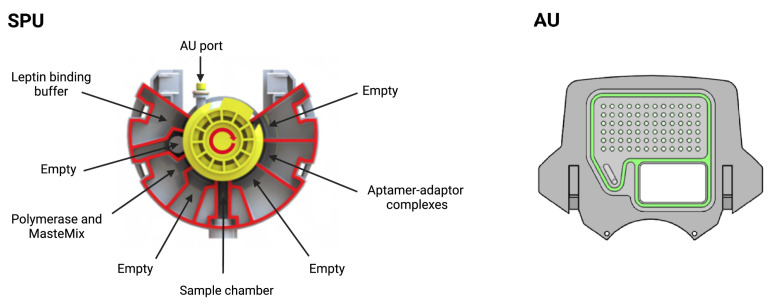
Sample preparation unit (SPU) and amplification unit (AU). In the SPU, the empty compartments are used to collect waste or for additional buffers depending on the protocol. In the AU, all wells contain primers and several wells are used for the standards. Reprinted/adapted with permission from Ref. [12]. 2020, the Authors.

The cartridge, shown in Figure 4, is an adapted version of our DnaCartridge described in [12]. It is a disposable, sealed and integrated lab-on-chip device that allows sample-to-result qPCR. It consists of two main parts, the amplification unit (AU) and the sample preparation unit (SPU), as depicted in Figure 5.

**Figure 6:**
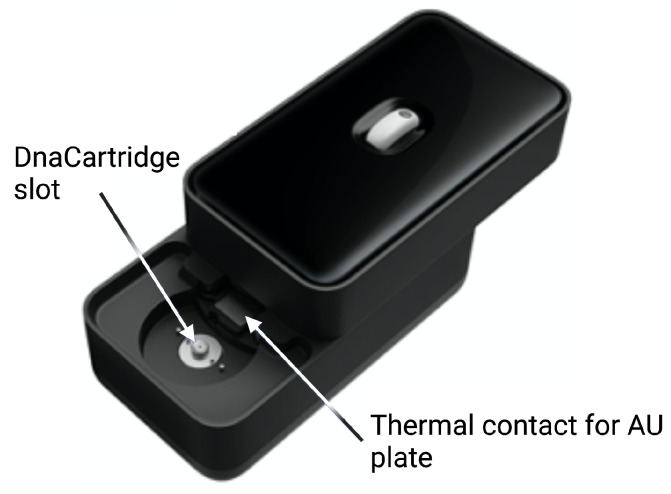
External case of the POC device. Reprinted/adapted with permission from Ref. [12]. 2020, the Authors.

The SPU consists of circumferentially distanced chambers around a rotatable mixing chamber (Figure 5, top). The chambers contain the aptamer–adaptor complexes, the leptin binding buffer, the polymerase and the dried qPCR MasterMix (without the primers, which are deposited in dry format the into AU’s wells). The empty compartment are used to collect waste or for additional buffers depending on the protocol, which highlights the versatility of the cartridge. The mixing chamber in the middle fits onto a rotating spigot on the NudgeBox (Figure 6), with a pneumatic port that helps moving reagents between the middle chamber and the rest. The DNA extraction is performed by applying pressure through a syringe. The AU (Figure 5, bottom) contains the primers that are spotted in nanolitres into the wells and air-dried. Then, to provide replicates and increase accuracy, they are distributed into several wells.

The sample preparation depicted in Figure 3 happens in the SPU, which is preloaded with aptamer–adaptor complexes (Figure 3A1). After sample loading in the swab chamber, the leptin in the sample displaces the complexes due to high affinity with the aptamers. Subsequently, the solution is mixed with the leptin binding buffer, which enhances the complex-displacing process. During this step, the solution is incubated at 37 ${}^{\circ }$C for 30 min (Figure 3A2). The solution is then mixed and incubated with the qPCR MasterMix and the polymerase 60 ${}^{\circ }$C for 10 min, which extends the undisplaced complexes to form fully double-stranded sequences (Figure 3A3). Finally, the solution is transferred to the AU via the AU port, where the solution is mixed with the primers, which are designed to bind specifically to the double-stranded sequences without amplifying free adaptors or aptamers (Figure 3A4). The AU also contains the wells with the leptin standards used for calibration. The AU is placed on a thermal plate (Figure 6), which enables qPCR amplification.

### 3.4. qPCR Module

Similar to other conventional qPCR machines, this module comprises a heating and a cooling element that enable the thermal cycling for the qPCR; however, unlike usual qPCR machines, our module must include a temperature profile for incubation with sample and with master mix during the sample preparation (see Figure 3A3). After sample preparation, the thermocycler provides the thermal conditions required by the qPCR, i.e., pre-incubation at 95 ${}^{\circ }$C for 600 s and a three-step qPCR amplification: DNA denaturation 95 ${}^{\circ }$C for 10 s, primer annealing at 60 ${}^{\circ }$C for 10 s and DNA elongation at 72 ${}^{\circ }$C for 10 s. At each annealing step, the blue light of a given wavelength produced by the LEDs (λ≈497 nm) is absorbed by the SYBR green dye, which emits fluorescence proportional to the target DNA concentration in the chambers. The photodiodes and readout circuit capture the green light (λ≈520 nm) emitted by the dye.

The design of the qPCR machine has a significant impact on the quantification performance. For example, the speed of the thermal cycling affects the efficiency of the DNA amplification, and noise in the fluorescence detection affects measurement precision [36].

### 3.5. Real-Time qPCR Data Processing

The fluorescence data contain the noise introduced during the biochemical process—due to mixing faults and inefficiencies in the biochemical process—named ProcessFluorescence. We name BackgroundFluorescence the noise introduced during the qPCR due to device performance and reaction efficiency, and during the fluorescence reading, due to noise introduced by fluctuations in photodiodes and LED light intensity. The lack of washing steps simplifies the device, which would otherwise require immobilising the aptamers on the wells’ surface, a washing buffer and additional hardware to mix and discard the waste solution. As a result, the free sequences and proteins remain in the chambers and can interfere with the qPCR. A negative control is included in the assay and subtracted from the standards and samples to reduce the noise due to the biochemical process (ProcessFluorescence). Additionally, the control serves as a validation signal for the assay, since it provides a limit on the maximum number of complexes that can be detected. In fact, if the signals produced by the unknown samples show earlier amplification than the negative control, then the free sequences have interfered with the amplification. Consequently, the cartridge is discarded as invalid and the test is repeated.

**Figure 7:**
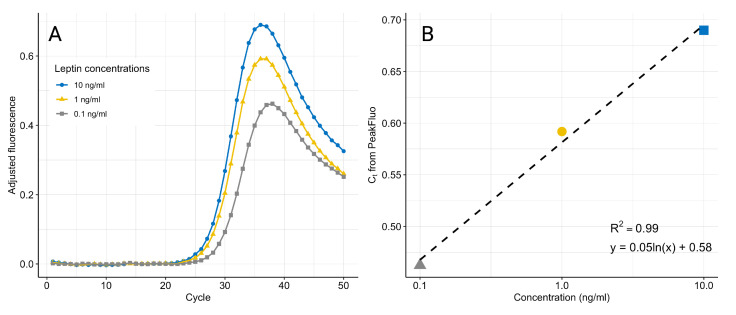
qPCR fluorescence amplification curves after removal of BackgroundFluorescence and ProcessFluorescence (**A**). The noise-adjusted signals decrease at higher cycles as the amplification curves reach a common plateau. The maximum fluorescence at the peak is used to generate the standard curve (**B**). The data are an example from one experiment using three replicates for each concentration.

We have developed a new algorithm, PeakFluo (see Algorithm 1), to process the fluorescence signal in real-time while the qPCR is running. BackgroundFluorescence represents the background fluorescence produced by the solution in the chamber and due to device performance. The background fluorescence is detectable when the number of target DNA copies is not enough to produce a visible signal, and therefore BackgroundFluorescence is determined as the average fluorescence in the first 10 cycles. From cycle 11, at each cycle, the BackgroundFluorescence is subtracted from all signals including the control, and the process noise ProcessFluorescence is removed from the standards and unknowns. Given the convex nature of the cleaned fluorescence signal (see Figure 7), the process stops once the global minimum is found for each sample, thus reducing the qPCR running time. For a more straightforward interpretation, the standard curve is generated from the absolute value of the global minimum. The instrument performs the qPCR and calculations in real-time [12], and a standard curve to quantify the samples is generated by the same algorithm ran on the standards included in the cartridge.

Including replicates and taking the average fluorescence across them is an easy and effective way to minimize the noise due to LED and photodiode variation.
**Algorithm 1** PeakFluo: self-calibrating algorithm for early-cycle protein quantification1:**procedure**Find chamber noise($BackgroundFluorescence_{n}$)2:    **for** c=1 to 10 **do**3:        $BackgroundFluorescence_{n}\leftarrow \max\left(f_{n}\left(c\right)\right)$      ▹ For n=0,...,N chambers4:    5:**procedure**Find peak fluorescence($peak_{n}$)                         ▹ For n=0,...,N chambers6:    $peak_{n}=0$7:    **while** foundPeak = False **do**8:        **for** c=11 to *C***do**                                                                                                        ▹*C* is total cycles9:                $f_{n}{\left(c\right)}_{clean}\leftarrow f_{n}\left(c\right)-BackgroundFluorescence_{n}$10:                $f_{n}{\left(c\right)}_{clean}\leftarrow f_{n}\left(c\right)-ProcessFluorescence\left(c\right)$ ▹$ProcessFluorescence\left(c\right)=f_{0}\left(c\right)$11:           **if** $f_{n}{\left(c\right)}_{clean}<{\mathrm{peak}}_{n-1}$ **then**12:               $peak_{n}\leftarrow \left|f_{n}{\left(c\right)}_{clean}\right|$13:           **else**14:               foundPeak = True15:      16:**procedure**Quantify sample($conc_{sample}$)17:    $m=\frac{peak_{c1}-peak_{c2}}{conc_{c1}-conc_{c2}}$                                                                                         ▹$c_{1}$ and $c_{2}$ are the standards18:    $conc_{sample}=\frac{peak_{sample}-peak_{c2}}{m}+conc_{c1}$

### 3.6. Convolutional Neural Network for qPCR Data Classification

A convolutional neural network is trained and validated on 278 qPCR curves generated from the amplification of samples containing leptin standards in various concentrations (0.1, 1, 10 ng/mL). Leptin concentrations are manually labelled into two classes—“high” or “low”—depending on a threshold of 5 ng/mL, which was chosen for its clinical significance. In fact, while leptin-resistant obesity is characterised by increased leptin levels, leptin-deficient obesity is characterised by leptin levels below 5 ng/mL [37]. The dataset composed of 278 qPCR images was collected during the experiments to validate the leptin aptasensor. A total of 201 curves were generated from leptin standards higher than 5 ng/mL and 77 from leptin standards lower than 5 ng/mL.

**Figure 8:**
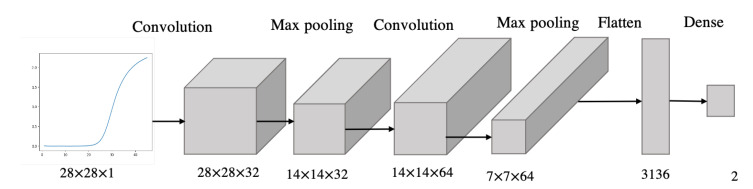
Architecture of the CNN model.

We trained a 6-layer CNN, composed of a convolution layer, max-pooling layer, convolution layer, max-pooling layer, flatten layer and dense layer (Figure 8). The inputs to the CNN are the fluorescence curve images, while the outputs are the labels “high” or “low”, classifying the leptin concentrations based on the preset threshold. The model was validated with stratified k-fold cross-validation to compensate for the class imbalance. Performance was assessed through classification accuracy and loss.

### 3.7. Samples for Methods Development and Validation

The biochemical assay was developed and validated with human salivary leptin. Details of the validation methods can be found in [18]. PeakFluo and the CNN were trained and validated on leptin standards quantified through the biochemical assay. The qPCR fluorescence signals were obtained with a commercially available PCR machine.

## 4. Results

### 4.1. Biochemical Assay

At the core of the point-of-care device, there is our novel method to quantify leptin with aptamers, which was developed as described in [18]. In the method, the leptin concentration is inversely proportional to the number of leftover complexes. Adaptors and aptamers in concentrations of 330 nM and 3.3 nM, respectively, were incubated for 15 min at 37 ${}^{\circ }$C and then incubated with leptin standards in concentration 10 ng/mL, 1 ng/mL and 0.1 ng/mL. All samples were ran in triplicates. As the targets of amplification are the complexes that were undisplaced by leptin, the $C_{t}$ value is directly proportional to the leptin concentration. In fact, if the leptin concentration is higher, there will be less leftover complexes and thus later amplification. Furthermore, the negative control (0 ng/mL leptin sample) is amplified earlier than the samples containing leptin since it is the sample for which no complexes have been displaced.

Primer specificity was assessed through uMELT package, which predicted a $T_{m}$ value of 84 ${}^{\circ }$C, and was further demonstrated by a single peak in the melting curve analysis with the same $T_{m}$ value.

The assay was validated with human leptin, and recovery was measured by spiking the saliva samples with synthetic leptin (for detailed results, please refer to [18]).

Table 2 provides a comparison between our assay and other assays developed for leptin quantification [18]. Our aptasensor reduces reaction times and automates sample preparation, all while using a very small sample volume.

### 4.2. Real-Time qPCR Data Processing

**Figure 9:**
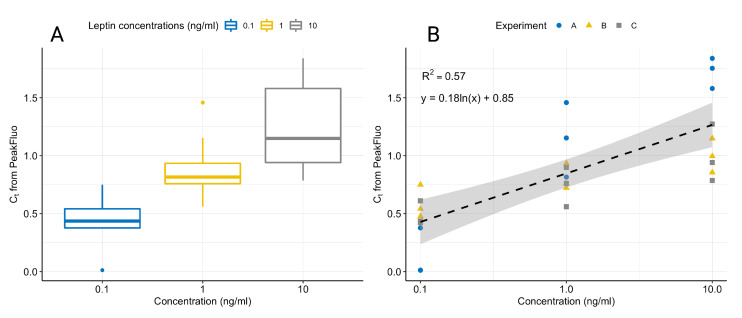
Boxplot showing the variation in normalised leptin concentrations for three experiments on separate days (**A**). Linear fit of normalised leptin concentrations for three experiments on separate days with 95% confidence intervals (**B**). All samples were ran in triplicates and data were normalised by dividing each $C_{t}$ value by the average $C_{t}$ value for all concentrations in a given experiment.

We collected the fluorescence data with a commercially available qPCR machine, using samples prepared manually with the proposed assay. Figure 7 displays the fluorescence signals after the removal of ProcessFluorescence and BackgroundFluorescence, showing the characteristic peak. The standard curves generated by fitting a linear model to the log(concentration) and the peak fluorescence produced by PeakFluo achieved better $R^{2}$ than the standard curves fitted on the $C_{t}$s estimated by the commercial software. All samples were ran in triplicates, and the results were replicated across several experiments (see Table 3 and Figure 9). From Figure 9, it can be seen that we can reliably detect concentrations in the 0.1–10 ng/mL range, and that our lowest limit of detection is 0.1 ng/mL.

PeakFluo improves linearity by removing the residual fluorescence introduced by the leptin, aptamers and free adaptors remaining in the solution, because they could interfere with the qPCR. This residual fluorescence can be removed by including a negative control, thus improving the precision of the measurement. The algorithm also reduces the time-to-results, as it allows the qPCR to stop around cycle 35 (see Figure 7), thus reducing the time-to-result by approximately 20 min. Finally, the PeakFluo method operates in real time and it is easily implementable on a portable device.

### 4.3. Convolutional Neural Network for qPCR Data Classification

Alongside providing quantitative measurements of protein concentrations, our device can be employed in alternative ways that can provide clinically relevant advice. As discussed in Section 2.2, conventional techniques for the analysis of qPCR data involve examining only parts of the fluorescence curve (for example, the exponential phase) to extract relevant features (for example, the C${}_{t}$ value); however, the other phases of the fluorescence curve could hold other information useful to identify target concentrations and differentiate between samples and controls. Therefore, we propose an alternative way of analysing the whole fluorescence curve as an image by utilising convolutional neural networks. In this way, we are able to analyse the complete curve instead of examining only one of its parts. Moreover, with this approach, we avoid the calibration steps needed to analyse qPCR data, such as background removal, calibration and generation of the standard curve.

**Figure 10:**
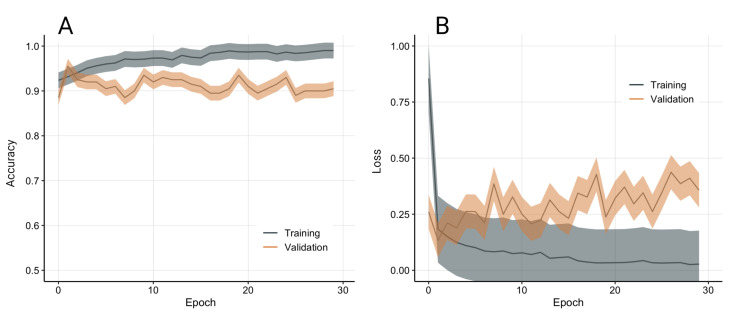
Model accuracy (**A**) and loss (**B**) from 10-fold stratified cross-validation. Data presented as average and standard deviation.

A total of 278 qPCR images were collected from a commercially available qPCR machine, and manually labelled in two classes based on a threshold of 5 ng/mL (201 high concentration, 77 low concentration). The model was validated with stratified 10-fold cross-validation to compensate for the class imbalance. The model achieved good training and validation performance (see Figure 10).

## 5. Discussion

This work presented a fully automated, portable and rapid system for sensitive protein quantification in a POC setting. The device is based on our novel aptamer-based method for protein quantification that achieves a limit of detection suitable for leptin quantification while using a lower sample volume and significantly less time compared to ELISA, which is the gold standard for protein quantification. Despite the limit of detection being higher than ELISA (100 VS 0.78 pg/mL), our work requires no hands-on time as the sample preparation is fully automated. Additionally, the proposed PeakFluo algorithm can significantly speed up the qPCR process, thereby reducing the time-to-result to under 2 h.

In this paper, we described the system’s modular design that includes sample preparation, DNA amplification and sensing without the need for human intervention. The disposable cartridge is designed for automated sample preparation and can be easily adapted to different buffers thanks to the empty compartments for waste disposal. The protein target is indirectly quantified through DNA amplification via qPCR, which is performed by the POC device. The PeakFluo algorithm described here allows for real-time quantification and calibration, which reduces the time-to-result.

Finally, we introduced a convolutional neural network trained on qPCR images produced by our device to classify samples based on leptin concentration. The relevance of this approach could be in the case of quickly classifying obese people with high or low leptin levels. In fact, while leptin is usually increased in obese patients and is proportional to adipose tissue, leptin is not correlated with BMI for people with the ΔG133 mutation in the *ob* gene, which encodes leptin [37]. Additionally, people with the ΔG133 mutation have significantly lower leptin levels compared to matched controls [37]. Consequently, to administer the right therapy for obese patients, one needs to know either their *ob* genotype or measure their circulating leptin levels. While the former is a viable option, its clinical usefulness is limited as genotypes are immutable. In contrast, monitoring leptin levels can provide ongoing information on the effectiveness of the therapy. We aim to develop the neural network further to improve its classification performance and to validate it on clinical samples, with the aim to provide valuable information to aid clinical decisions and improve outcomes for obese patients. A clinical trial will be needed to clinically validate the device for obesity classification, possibly by employing a three-arm design, including obese subjects with and without the ΔG133 mutation and non-obese subjects. Such a trial would also help assess the clinical usefulness of the device and better characterise its performance in terms of ease of use, speed and functionality.

This work presents some limitations. Firstly, despite representing a significant improvement compared to currently available ELISA assays, the time-to-result may still be considered long for a rapid POC device. Further development work will aim to shorten the device’s detection time. The computational methods, including PeakFluo and the CNN, have been tested on standard leptin concentrations and not on human samples. To validate the methods, tests on different types of human samples will be needed and will be the objective in future work.

## 6. Conclusions

This system represents the first POC device for leptin quantification, which can be easily adapted for the quantification of other proteins. Due to its modularity and versatility, our device is relevant to many applications, such as personalised healthcare (for example, by monitoring hormonal changes) and disease diagnostics using detection of protein biomarkers such as antigens and antibodies.

## Figures and Tables

**Table 1:** Oligonucleotide sequences used in the assay.

Name	Sequence (5’ to 3’)
Aptamer	GTTAATGGGGGATCTCGCGGCCGTTCTTGTTGCTTATACA
Adaptor	GCTACCCCGACCACATGAAGCAGCACGACTTCTTCAAGTCCGCCATGCCCGAAGGCTACGTCCAGGAGCGCACCATCTTCTTCAAGGACGACGGCAACTAAAAAATGTATAAGCAACAAGAACGGC
Complex	GCTACCCCGACCACATGAAGCAGCACGACTTCTTCAAGTCCGCCATGCCCGAAGGCTACGTCCAGGAGCGCACCATCTTCTTCAAGGACGACGGCAACTAAAAAATGTATAAGCAACAAGAACGGCCGCGAGATCCCCCATTAAC
Forward primer	CACATGAAGCAGCACGACTT
Reverse primer	TGGGGGATCTCGTGGC

**Table 2:** Comparison of leptin quantification methods. Reprinted/adapted with permission from Ref. [18]. 2021, American Chemical Society. (LoD: limit of detection; HoT: hands-on time; TtR: time to result).

Reference	Assay Type	LoD	K${}_{d}$	HoT ^*a*^	TtR	Sample
Imagawa, 1998 [38]	ELISA	0.78 pg/mL	83 pM	N/A	15 h +	100 μL
He, 2015 [39]	Chemiluminescent immunosensor	0.3 pg/mL	N/A	N/A	44 h +	100 μL
Tanaka, 2013 [40]	Waveguide-mode sensor	100 ng/mL	N/A	N/A	24 h +	400 μL
Dong, 2014 [41]	Electrochemical immunosensor	30 pg/mL	N/A	N/A	8 h +	-
Cai, 2019 [42]	Electrochemical immunosensor	0.036 pg/mL	N/A	N/A	78 h +	-
Chen, 2010 [43]	Electrochemical immunosensor	10 ng/mL	N/A	N/A	13 h +	-
Ojeda, 2013 [44]	Electrochemical immunosensor	0.5 pg/mL	N/A	N/A	95 min +	50 μL
Commercial ELISA	ELISA	15.6 pg/mL	N/A	1 h 20 min	3 h	10, 100 μL^*b*^
This work	Optical (qPCR) aptasensor	100 pg/mL	1.5 μM	0 min	<2 h	10 μL

*^a^* Hands-on time excludes incubation time and optical reading (the qPCR in the proposed methods takes 1 h),which are included in the time to result. *^b^* 10 μL for plasma and serum, 100 μL for supernatant.

**Table 3:** Comparison of the coefficient of determination $R^{2}$ achieved by PeakFluo and by the Roche LightCycle^®^ 96 software on three separate experiments.

Software	Exp. 1	Exp. 2	Exp. 3
PeakFluo	0.984	0.987	0.77
Roche LightCycler^®^ 96	0.384	0.973	0.53

## Data Availability

The data presented in this study are available on request from the corresponding author.
